# A Dual-Output Physiology-Informed Neural Network Architecture for Continuous Cuffless Blood Pressure Waveform Estimation: A Proof‑of‑Concept

**DOI:** 10.1007/s44200-026-00120-3

**Published:** 2026-08-01

**Authors:** Zaineb Aloui, Cederick Landry

**Affiliations:** 1 Department of Mechanical Engineering, Université de Sherbrooke, Sherbrooke, QC Canada; 2 Research Center on Aging, CIUSSS de l’Estrie–CHUS, Sherbrooke, QC Canada

**Keywords:** Arterial compliance, Cuffless blood pressure, Electrocardiography, Physiology-informed neural network, Photoplethysmography, Wearables

## Abstract

This pilot study introduces the first subject‑specific, physiology‑informed neural network approach for accurate estimation of the full blood pressure (BP) waveform. The method builds on a nonlinear autoregressive model with exogenous inputs (NARX), implemented through artificial neural networks trained on subject‑specific electrocardiography (ECG) and photoplethysmography (PPG) signals. Three model configurations are evaluated: (1) a reference NARX model trained on 30 min of data, (2) a reduced‑data NARX model trained on 15 min, and (3) a physiology‑informed model (NARX_physio_), also trained on 15 min, which incorporates a personalized four‑parameter BP–PPG sigmoidal layer into the training process through a dual‑output loss function that simultaneously estimates the BP and PPG waveforms. By embedding BP–PPG physiology directly into training, the architecture ensures internal physiological consistency—something not achieved in existing PPG‑based or purely data‑driven machine learning approaches. All three models were assessed on a small (*n* = 5), but rich dataset, covering over six hours of activities of daily living. Despite using only half the training duration of the baseline NARX model, the physiology‑informed approach maintains accurate BP estimation. Beyond improved accuracy, the sigmoidal layer provides physiologically interpretable parameters, including estimated arterial‑compliance curve width with an average of 36.5 mmHg and an estimated PPG contact pressure with an average of 59 mmHg in this study, offering insights unavailable in standard neural network models. Together, these preliminary results suggest that the NARX_physio_ model enables effective personalization of cuffless BP waveform estimation with limited training data, highlighting its potential to improve both the reliability and the clinical relevance of continuous cuffless BP monitoring.

## Introduction

Hypertension, or high blood pressure (BP), accounts for roughly 19% of deaths worldwide [1]. Even at a systolic BP (SBP) as low as 130 mmHg—well below the historical threshold for hypertension—the risk of heart disease rises dramatically, increasing by 81% [2] compared to a SBP of 100 mmHg. Despite this known risk, only 21% of hypertensive patients achieve satisfactory therapeutic control, while nearly 46% remain undiagnosed [3]. The gap between the level of clinical risk and current patient monitoring reflects the lack of appropriate technologies. Ambulatory BP monitoring, 24 h monitoring with an arm cuff every 20–30 min, is considered a reference method due to its diagnostic accuracy and reliable measurements [4]. Yet, because of discomfort and practical constraints of the cuff for patients, it remains impractical for prolonged daily use. In this context, cuffless BP monitoring devices offer a promising alternative, enabling continuous, unobtrusive tracking that could improve long-term cardiovascular risk management [5].

Among cuffless methods, pulse wave analysis (PWA)—often combined with pulse arrival time (PAT)—is the most widely used and is likely the only approach implemented in regulatory-cleared devices [6, 7]. These PWA BP estimation models typically rely on photoplethysmography (PPG), which reflects distal blood volume changes, or tonometry, which captures peripheral arterial forces, to large datasets to learn the relationship between the extracted PWA features and SBP and diastolic BP (DBP) values [8]. These PWA models are generally easily integrated into wearable devices since they require only a one-time or periodic cuff calibration.

However, recent studies [9–11] have raised concerns about the ability of population-based PWA models to reliably track BP changes after cuff calibration. This may be because the PPG signal reflects multiple subject-specific physiological influences beyond BP (e.g., vascular tone, arterial stiffness, and peripheral circulation). A central assumption in the present work is that subject-specific models are essential for achieving accurate cuffless BP estimation. Although few studies have directly compared subject-specific and cross-subject models, at least one investigation using PWA with bioimpedance demonstrated improved performance with a small number of participants [12].

Subject-specific models have long been central to PAT and pulse transit time (PTT) approaches [8]. These models typically require only one to a few BP measurements for calibration. However, due to their simplicity and various limitations, they may not perform well in tracking BP accurately across different conditions, though they may be useful for screening for hypertension [13]. Similarly, simple subject-specific PWA models with few parameters have been developed and require minimal training data [14]. Yet, as model complexity increases to improve accuracy, the number of required BP measurements to train the subject-specific model also rises—sometimes up to a full day of data collection [15].

While most studies have focused on estimating SBP and DBP [8], some have explored modeling the complete BP waveform. This approach may offer advantages for model training, as it provides a richer set of informative data points beyond just SBP and DBP. Moreover, the full waveform can yield additional insights into cardiovascular dynamics, such as cardiac output and vascular resistance [16]. To enable BP waveform reconstruction from physiological signals, few model architectures have been proposed. Among these, a Conv1d-BiLSTM neural network using PPG, ECG, the first and second derivatives of the PPG signal [17], and a 1 d U-Net architecture designed to reconstruct the full waveform from PPG alone [18] were proposed. SBP and DBP values are then extracted from the complete BP waveform.

To the authors’ knowledge, the only subject-specific approach to estimate the complete BP waveform uses nonlinear autoregressive models with exogenous inputs (NARX), which have demonstrated promising results when using both PPG and ECG signals compared to PPG alone [19]. This method also demonstrated the ability to track large BP changes during daily activities [20]. However, this NARX model required complex subject-specific training data including two 15-minute segments (at the start and at the end of the estimation period), including phases of physical activity to capture a wide range of subject-specific BP variations, potentially limiting clinical adoption and the estimation span where the model remains accurate.

In this study, building on this previous body of work, we propose a dual-output physiology-informed neural network architecture to reduce the amount of data required for subject-specific model training, i.e., only using the short 15-minute window recorded at the beginning of the estimation period. Minimizing the data requirements for model training is vital, as it enhances the clinical feasibility of implementing these high accuracy subject-specific models in real-world settings. Our proposed physiology-informed approach combines BP estimation with the reconstruction of a PPG signal, which requires the estimated full BP waveform, in order to introduce a physiological BP-PPG sigmoidal relationship constraint during training. Unlike conventional single‑output neural networks that estimate only BP, our architecture produces two coupled outputs—BP and PPG—allowing physiological constraints to directly influence the learning dynamics. The main contributions of this work are as follows:

- A novel physiology‑informed dual‑output architecture that simultaneously estimates the BP waveform and a physiologically constrained PPG waveform by embedding a personalized BP–PPG sigmoidal model directly into network training.
- A pilot demonstration that physiological constraints can reduce subject‑specific training data requirements by half, while maintaining or improving BP waveform estimation accuracy.
- An interpretable physiological neural network layer that provides parameters (e.g., compliance‑curve width), which potentially provides insight into arterial stiffness.

## Methods

### Experimental Procedure

The data under analysis were collected as part of a previously published protocol [20] and are available on IEEE DataPort [21] (see Data Availability Section), and approved by the University of Waterloo Ethics Committee (ORE#41490). The study involved five healthy adults (four males, age 28 ± 6.6 yrs) of varied fitness levels, ranging from sedentary to regularly active, and free of cardiovascular and peripheral vascular disease who were monitored continuously for approximately 6.5 h. The protocol included two 15-minute standardized sequences (at the start and at the end of the monitoring period) featuring various body postures and physical activities (static handgrip, Valsalva maneuvers and walking) to rapidly cover a large range of BP and heart rate, as well as a 6-hour period of daily activities. During the data collection period, Astroskin wearable body metrics vest (Carré Technologies Inc., Canada) forehead PPG and ECG, plus Finapres^®^ BP were collected at 64 Hz. For more details on the complete data collection protocol, see Landry et al. [20].

This dataset is notably distinct from typical publicly available datasets. It provides continuous waveform signals during real-world daily activities, something nowhere else captured due to the technical difficulty of measuring the BP waveform. It was acquired using the Portapres^®^ device, a now-discontinued system by Finapres^®^, making replication of such data collection extremely difficult. To our knowledge, no other dataset offers this combination of signals for such a long period of time in real-life settings. While the dataset includes only five participants, it nonetheless provides a valuable platform for evaluating the feasibility of our physiology‑informed dual‑output architecture for real-life cuffless BP measurement.

### Model Architecture

#### Baseline NARX Model: NARX

 Inspired by the single-layer NARX architecture described in a previous article [20], a NARX model was implemented to estimate the instantaneous BP waveform. The network was composed of three hidden layers with 32, 16, and 8 neurons, each using the rectified linear unit (ReLU) activation function. A linear output layer produced the BP estimation ($\:\widehat{BP})$ one time step ahead. At each time step, the input vector was formed by concatenating the 38 most recent samples of PPG and ECG signals, along with the last four estimated BP values. During training, the measured BP values were used as input instead. The model was trained for 200 epochs with a batch size of 64, with all input signals Min-Max normalized to the [0, 1] range, using Adam optimizer (with learning rate = 0.001), to minimize a loss function that equally combined the mean squared error (MSE) and mean absolute error (MAE). An early stopping procedure using 15% random split of the training data was applied to prevent overfitting. This model was selected as the baseline architecture as it represents, to our knowledge, the only subject-specific framework previously demonstrated to reconstruct the full BP waveform under dynamic daily living conditions. This choice enables a direct evaluation of the proposed physiology-informed extension while building upon an established and validated approach, shown to outperform simpler PAT models [19, 20].

#### Physiology-Informed NARX Model: NARX_physio_

**Fig. 1:**
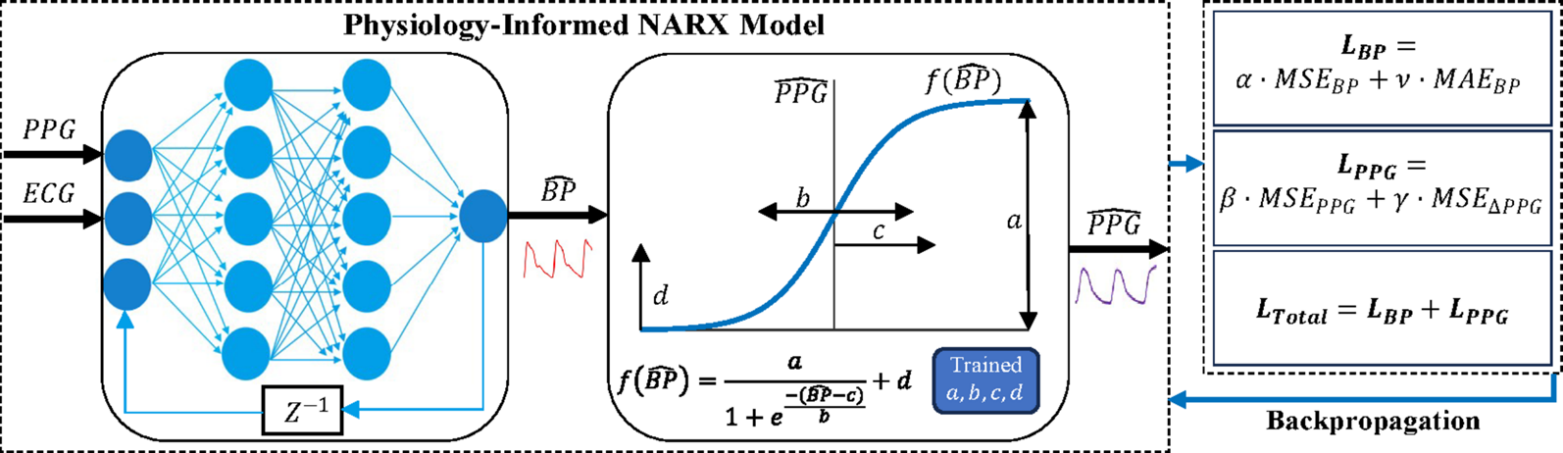
Schematic of the dual-output NARX_physio_ model: (1) The NARX model estimates BP from input ECG, PPG and BP sequences. (2) The estimated BP, ($\:\widehat{\boldsymbol{B}\boldsymbol{P}})$ is used as input to a parametric sigmoid layer with four trainable parameters (*a*,* b*,* c*,* d*) to estimate the PPG, ($\:\widehat{\boldsymbol{P}\boldsymbol{P}\boldsymbol{G})}$. 3) A composite loss function (L_Total_) is used to update both models. It consists of a combination of BP and PPG losses (L_BP_, L_PPG_). The loss function coefficients were the same for all participants: $\:\boldsymbol{\alpha\:}$ = $\:\boldsymbol{\nu\:}$ = 0.5, $\:\boldsymbol{\beta\:}$ = 0.05, and $\:\boldsymbol{\gamma\:}$ = 0.005. Therefore, L_PPG_ represents about 5% of the L_Total_.

 NARX_physio_ was built from two complementary sub-models: the first is the standard NARX model, which estimates BP from ECG and PPG signals; the second integrates a parametric sigmoidal layer to generate an estimation of the PPG signal from this estimated BP (see Fig. 1).

This modeling approach draws on cardiovascular physiology: when BP increases, it dilates the arteries and increases the blood volume within, which is measured by the PPG sensor [22]. This nonlinear sigmoidal curve is a characteristic of the relationship between the transmural pressure of an artery and its blood volume within [18]. Herein, the model’s sigmoidal layer reproduced this BP-PPG relationship using the basic sigmoid function parameterized with four personalized parameters (*a*,* b*,* c*,* d*), which were initialized with small positive values (0.1, 0.2, 0.3, 0.1) to improve numerical stability during the early stages of training. This choice helps prevent premature saturation of the sigmoid function and supports smoother gradient propagation. Although different initialization schemes can affect convergence behavior, the parameters are fully optimized through backpropagation on each participant’s data. The estimated PPG signal ($\:\widehat{PPG})\:$was thus generated from the estimated BP $\:\left(\widehat{BP\:}\right)\:$ensuring an explicit physiological relationship between the two signals. The complete model therefore produced two outputs: one for BP estimation and one for PPG estimation. Importantly, during training, both outputs backpropagate through a shared loss, meaning the reconstructed PPG waveform directly influences BP estimation by enforcing a physiologically meaningful BP–PPG relationship. As shown in Fig. 1, NARX_physio_ training relied on a loss function that combines classical errors (MSE and MAE) of BP estimation and additional physiological terms assessing the shape and regularity of the PPG estimation (MSE of the estimated PPG and MSE of the PPG first-order derivative [23]). The loss function coefficients used in the following equations were the same for all participants (α = µ = 0.5, β = 0.05, and γ = 0.005).

#### Classical Loss

 The BP loss function combines MSE and MAE:

1 $$\:{L}_{BP}=\alpha\:\cdot\:MS{E}_{BP}+\nu\:\cdot\:MA{E}_{BP},$$

where,

2 $$\:\:MS{E}_{BP}=\frac{1}{N}{\sum\:}_{k=1}^{N}{\left(\widehat{BP}\left(k\right)-BP\left(k\right)\right)}^{2},$$

3 $$\:\:MA{E}_{BP}=\frac{1}{N}{\sum\:}_{k=1}^{N}\left|\widehat{BP}\left(k\right)-BP\left(k\right)\right|.$$

The loss function coefficients $\:\alpha\:$ and $\:\nu\:$ were the same for all participants and equal to 0.5.

#### Physiological Loss

 The PPG loss function combines MSE and derivative terms,

4 $$\:{L}_{PPG}=\beta\:\cdot\:MS{E}_{PPG}+\gamma\:\cdot\:MS{E}_{{\Delta\:}PPG},$$

with,

5 $$\:MS{E}_{PPG}=\frac{1}{N}{\sum\:}_{k=1}^{N}{\left(\widehat{PPG}\left(k\right)-PPG\left(k\right)\right)}^{2},$$

6 $$\:MS{E}_{{\Delta\:}PPG}=\frac{1}{N}{\sum\:}_{k=1}^{N}{\left(\nabla\:\widehat{PPG}\left(k\right)-\nabla\:PPG\left(k\right)\right)}^{2}.$$

The loss function coefficients $\:\beta\:$ and $\:\gamma\:$ were the same for all participants and equal to 0.05 and 0.005, respectively.

The above loss term computes the MSE between the first-order derivatives of the estimated and measured PPG signals. The numerical derivative $\:\nabla\:PPG\left(t\right)=PPG\left(k+1\right)-PPG\left(k\right)$ represents the local slope of the waveform, enforcing similarity in waveform dynamics. This improves the physiological realism of the estimated PPG.

Then, the total loss is given by:

7 $$\:\:{L}_{Total}={L}_{BP}+{L}_{PPG},$$

which ensures both accurate BP estimation and physiologically consistent PPG estimation.

The MSE terms helped correct large errors quickly, while the MAE term made the model more robust to noise and outliers. This combination improved gradient flow and led to better convergence during training. This complete architecture favoured a coherent estimation of BP while preserving the physiological compatibility with PPG personalized for each subject. To validate the contribution of the physiological loss terms, ablation studies were performed by removing either the $\:MS{E}_{{\Delta\:}PPG}$ or $\:MS{E}_{PPG}$ component from the loss function.

#### Baseline Mean Training Data Model: MTD

 Finally, a mean training data (MTD) was trained, which used the mean of SBP and the mean of DBP during the first 15 min of training data to estimate six hours of BP during daily life activities. MTD was used to simulate a one-time cuff measurement to estimate someone’s BP over six hours. This MTD model is used to demonstrate the added value beyond calibration/training of our cuffless BP model. Each model configuration was trained independently for every participant.

### Training

In the previous study [20], the entire 30-minute training dataset was used for model training (i.e. the first and last 15 min of the complete 6.5-hour dataset) independently for each participant. In this work, three configurations were tested with the proposed model: (1) using all 30 min to train the standard NARX (NARX_30min_) as a baseline comparison, (2) using only the first 15 min to train the NARX model (NARX_15min_) to assess the effect of reduced training data, and (3) using the first 15 min to train the NARX_physio_.

### Signal Preprocessing

To ensure reliable inputs for model training and evaluation, a signal validity pipeline was applied to the PPG, ECG, and BP recordings. The ECG and PPG signals were already preprocessed through the Astroskin vest’s proprietary filtering algorithms, and the BP waveform was left unfiltered. Quality assessment was performed using visual inspection, with particular attention to the BP signal, as it served as the reference for model performance evaluation. Segments presenting clear artifacts or corrupted waveforms were excluded. All removals were executed synchronously across the three signals to maintain strict temporal alignment. During training, only segments deemed valid for all signals were retained to ensure clean input–output mappings. During prediction, no segments were excluded a priori in order to emulate realistic operating conditions where artifacts may naturally arise. Instead, invalid segments identified post hoc were removed only prior to the computation of performance metrics.

### Performance Evaluation

For all three model configurations, the performance was evaluated on six hours of unseen daily activity data across the five participants. Each model produced a continuous BP waveform, from which SBP and DBP were extracted beat by beat. To benchmark the different models, four quantitative performance indicators were computed beat by beat for SBP and DBP: the MAE, which provides an overall indication of estimation accuracy, the Pearson correlation coefficient (*r*), quantifying the linear relationship and the similarity of temporal dynamics between the estimated and measured signals, as well as the mean error (µ_Err_) and the error standard deviation (σ_Err_). The computed metrics were averaged across participants and were displayed using bar plots to compare the estimation performance of the four models: MTD, NARX_30 min,_ NARX_15min_ and NARX_physio_. Each performance metric was then compared using Wilcoxon rank sum tests (α = 0.05) as Wilcoxon signed rank test is limited to a p-value of 0.0625 for a sample size of 5. Bonferroni corrections were applied to identify any statistical differences between the models.

To assess potential temporal drift, a 20-minute moving average of the estimation error was computed over the 6-hour monitoring period for all models.

To assess the impact of activity and motion context on model performance, the test dataset was divided into three subsets: (i) sitting, (ii) standing, and (iii) walking. Walking segments were identified using the vertical accelerometer signal from the Astroskin wearable body metrics vest, based on body motion and deviation from the gravity vector. Sitting and standing segments were distinguished using orientation information derived from the phone angle measurement. This segmentation allows for evaluating model performance under different activity conditions.

The $\:\widehat{PPG}\:$ signal was generated exclusively by the NARX_physio_ model using the estimated BP, relying on the explicit physiological relationship between the two found during training. Both $\:\widehat{PPG}$ and $\:\widehat{BP}\:$ were then compared to their measured signals to evaluate signal agreement through visual inspection of the waveform shapes. MAE of the complete BP waveforms to assess its estimation accuracy. The PPG MAE was normalized by the standard deviation of the measured PPG signal over the recording period. Thus, a normalized MAE of 1 corresponds to an error equal to the typical amplitude of signal variability.

### Physiological Parameters Extraction

In the NARX_physio_ model, the parameters *b* and *c* are of particular interest, as they respectively characterize the width of the artery compliance curve and the PPG contact pressure. However, within the NARX_physio_ neural network architecture, the input and output values are normalized using Min-Max normalization, such that the following sigmoid function (introduced in Fig. 1) allows for modeling the relationship between the normalized BP ($\:{BP}_{n}$) and the normalized PPG ($\:{PPG}_{n}$):

8 $$\:\:\:\:{PPG}_{n}=\frac{{a}^{\:}}{1+{e}^{-\left({BP}_{n}-{c}^{\:}\right)/{b}^{\:}}}+{d}^{\:},$$

where,

9 $$\:{BP}_{n}=\frac{BP-{BP}_{Min}}{{BP}_{Max}-{BP}_{Min}}\:\:\:\:\:\:\:,\:\:{PPG}_{n}=\frac{PPG-{PPG}_{min}}{{PPG}_{max}-{PPG}_{min}}$$

The parameters *c* and *b* correspond to the abscissa offset of the inflection point (at 50% level) and the width of the sigmoid, respectively, while *a* denotes the maximum amplitude of the sigmoid, and *d* the ordinate offset.

The original PPG values can then be mapped back to their physiological scales by applying the inverse Min-Max normalization applied to the model variables:

10 $$\begin{gathered} \:PPG = \:\:\left( {PPG_{{max}} } \right. \hfill \\ \left. { - PPG_{{min}} } \right)\left( {\frac{{a^{\:} }}{{1 + e^{{ - \left( {\frac{{BP - BP_{{Min}} }}{{BP_{{max}} - BP_{{Min}} }}\: - \:c^{{\kern 1pt} } } \right)/b\:^{{\kern 1pt} } }} }} + d^{\:} } \right) + \:PPG_{{min}} \hfill \\ \end{gathered}$$

This expression can be equivalently written in the standard sigmoid from:

11 $$\:g\left(BP\right)=PPG=\frac{{a}^{{\prime\:}}}{1+{e}^{-\left(BP-{c}^{{\prime\:}}\right)/{b}^{{\prime\:}}}}+{d}^{{\prime\:}}$$

With the transformed parameters obtained by direct identification, where (*c’*, *b’*) are derived from the denormalization of BP, and (*a’*, *d’*) from PPG denormalization:

12 $$\:{c}^{{\prime\:}}=\left({BP}_{Max}-{BP}_{Min}\right)c\:+{BP}_{Min}\:$$

13 $$\:{b}^{{\prime\:}}=\left({BP}_{Max}-{BP}_{Min}\right)b$$

14 $$\:\:{a}^{{\prime\:}}=\left({PPG}_{max}-{PPG}_{min}\right)a\:$$

15 $$\:{d}^{{\prime\:}}=\left({PPG}_{max}-{PPG}_{min}\right)d+{PPG}_{min}\:$$

The parameters *a’* and *d’* are not of physiological interest, as PPG is measured in volts rather than in units of volume, which would have reflected changes in arterial diameter. However, *b’ and c’* represents the width of the artery compliance curve and the PPG contact pressure in units of mmHg.

To obtain a comparable measure of the sigmoid width independently from the sigmoidal function type, the full width at half maximum (FWHM) of the derivative of $\:g\left(BP\right)$ was used. The derivative is given by:

16 $$\:{g}^{{\prime\:}}\left(BP\right)=\frac{{a}^{{\prime\:}}}{{b}^{{\prime\:}}}\frac{{e}^{-\left(BP-{c}^{{\prime\:}}\right)/{b}^{{\prime\:}}}}{{\left(1+{e}^{-\left(BP-{c}^{{\prime\:}}\right)/{b}^{{\prime\:}}}\right)}^{2}}\:.$$

This derivative has the shape of a bell curve, which reflects the compliance curve of the artery [24], with its maximum occurring at the inflection point $\:{c}^{{\prime\:}}$:

17 $$\:{g}^{{\prime\:}}\left({c}^{{\prime\:}}\right)=\frac{{a}^{{\prime\:}}}{4{b}^{{\prime\:}}}\:.$$

To characterize the width of $\:{g}^{{\prime\:}}$, we define the half-maximum points, $\:{x}_{\mathcal{l}}$ and $\:{x}_{r}$ obtained by solving:

18 $$\:\:\:\:{g}^{{\prime\:}}\left(x\right)=\frac{1}{2}{g}^{{\prime\:}}\left({c}^{{\prime\:}}\right).$$

The condition $\:{g}^{{\prime\:}}\left(x\right)=\frac{{a}^{{\prime\:}}}{8{b}^{{\prime\:}}}$, yields two symmetric solutions, which can be written as:

19 $$\:\frac{\left(x-{c}^{{\prime\:}}\right)}{{b}^{{\prime\:}}}=\pm\:k,\:k\approx\:1.76\:.$$

Thus, the half points are:

20 $$\:{x}_{\mathcal{l}}={c}^{{\prime\:}}-k{b}^{{\prime\:}},\:{x}_{r}={c}^{{\prime\:}}+k{b}^{{\prime\:}}.$$

The FWHM is then computed as the distance between these points:

21 $$\:FWHM={x}_{r}-{x}_{\mathcal{l}}=2k{b}^{{\prime\:}}\approx\:3.53{b}^{{\prime\:}}.$$

## Results

**Fig. 2:**
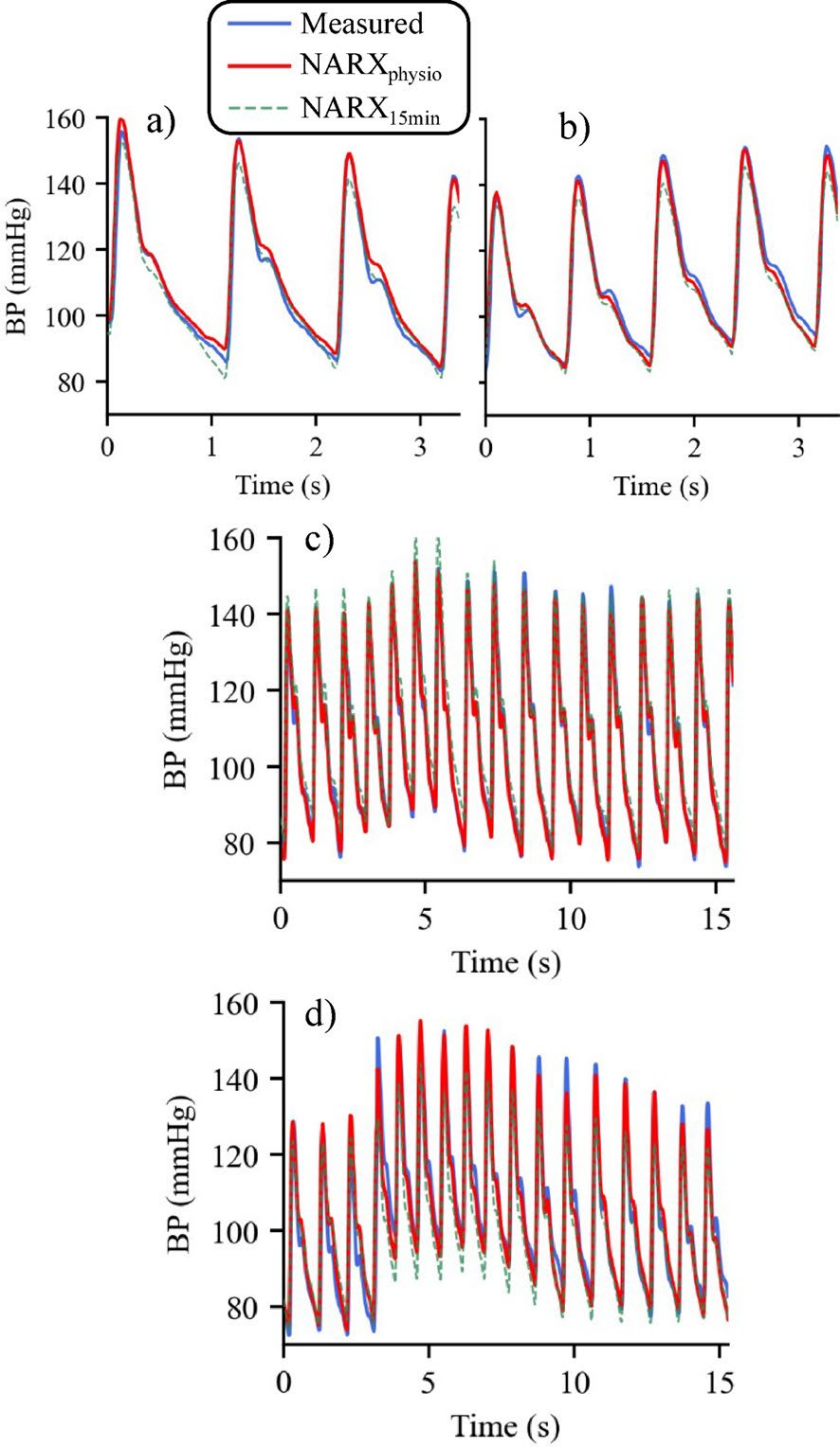
Comparison of the measured versus estimated BP waveforms from NARX_physio_ and NARX_15min_ for representative participants under BP variations. **a** and **b** shows BP increasing and decreasing for the same participant. **c** and **d** show BP variability for a second participant

Figure 2 shows the NARX_physio_ model’s ability to reproduce the BP waveform morphology in different scenarios. Figure 2 shows accurate estimation of systolic peaks and diastolic troughs. The MAE obtained with NARX_physio_ in Fig. 2a, b, c, and 2.d are 2.2, 2.3, 1.4, and 3.04 mmHg, respectively, showing a consistent reduction in error compared to 6.2, 5.1, 4.2, and 5.66 mmHg obtained with NARX_15min_ (not shown for readability). Figure 2d was selected to show the great performance in the NARX_physio_ ability to track rapid changes in BP.

**Fig. 3:**
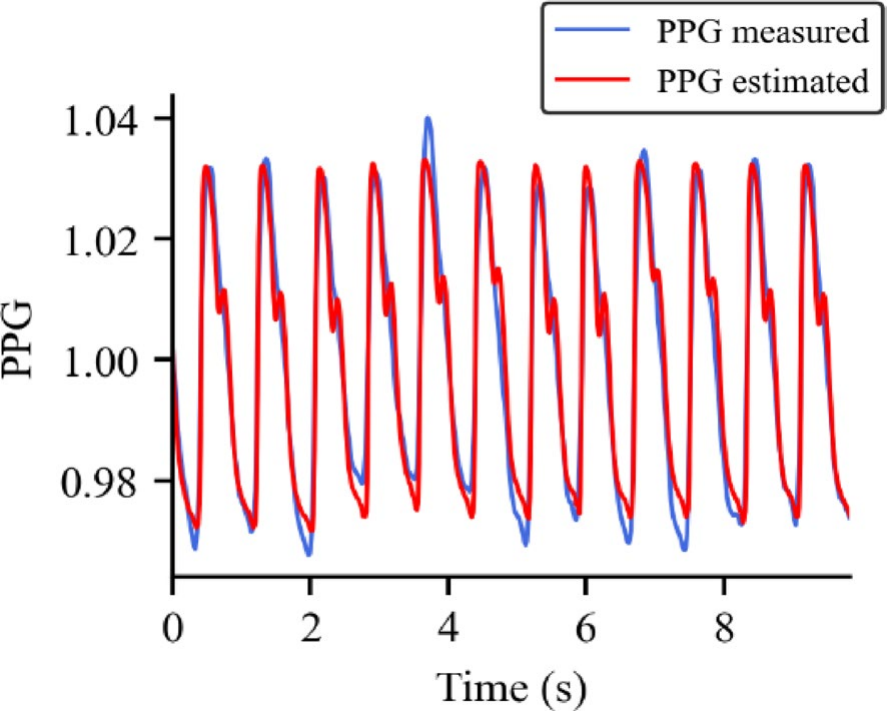
Comparison of the measured versus estimated PPG waveforms from NARX_physio_ for one representative participant

As illustrated in Fig. 3, the estimated PPG, generated by the NARX_physio_ model, exhibits a consistent morphology compared to the measured signal (normalized MAE = 0.4), even though a very simplistic 4-parameter sigmoidal model was used. The overall performance of the model to estimate PPG using the normalized MAE was 0.79 ± 0.20 when computed for all participants.

**Fig. 4:**
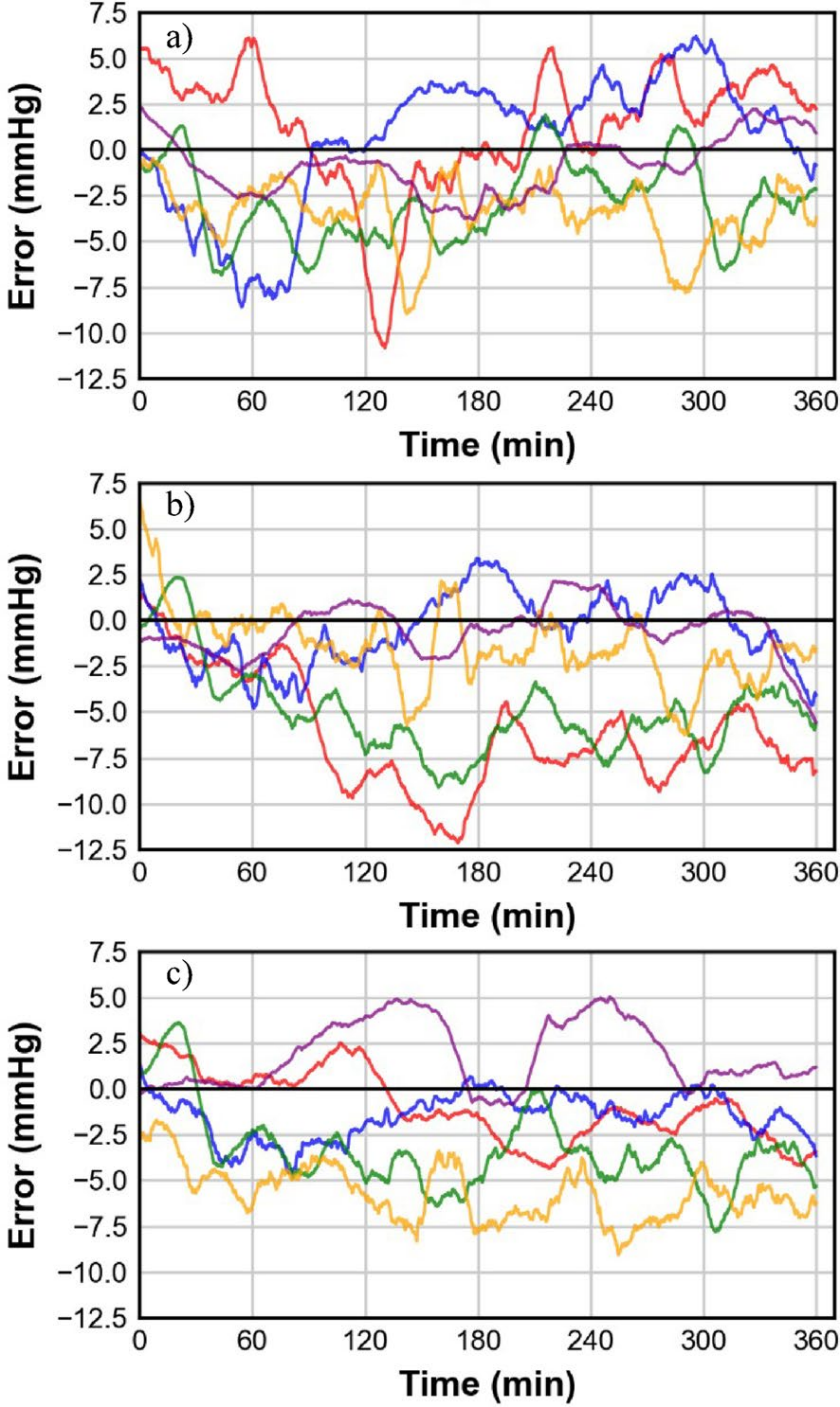
Temporal evolution of the BP estimation error for **a** NARX_30min_, **b** NARX_15min_, and **c** NARX_physio_ over the 6-hour monitoring period. Results are shown as 20‑minute moving averages to highlight low‑frequency trends. Each color represents a single participant

The temporal evolution of the estimation error over the 6-hour monitoring period is shown in Fig. 4 using 20-minute moving averages. The results indicate that the error remains bounded for all models, with no evidence of divergence. While some drift is observed, it varies across participants. In particular, the NARX_15min_ model tends to exhibit a negative bias over time, whereas the NARX_physio_ model remains more centered around zero, suggesting improved stability.

**Fig. 5:**
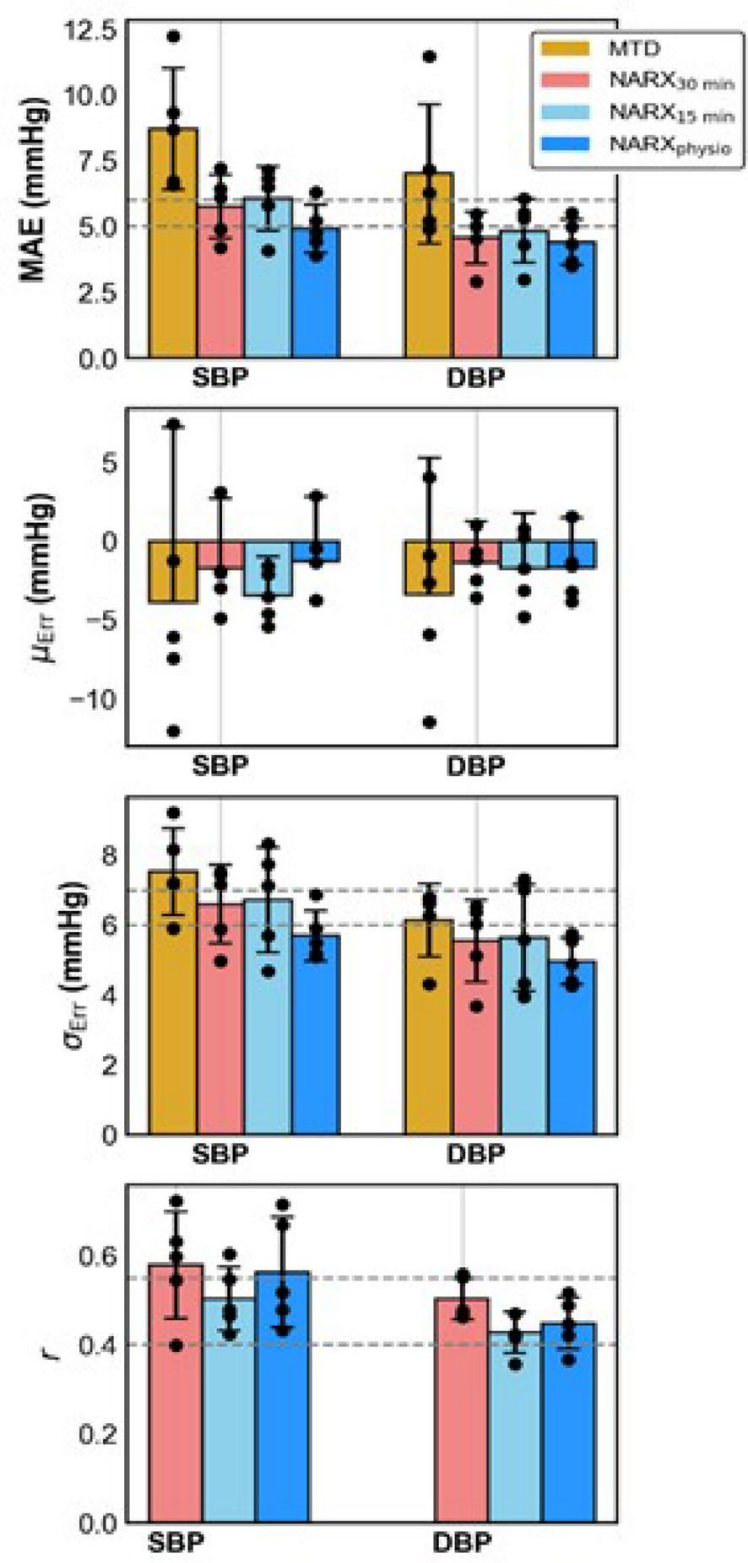
Comparison of mean absolute error (MAE), mean error (µ_Err_), standard deviation of the error (σ_Err_), and Pearson correlation (*r*) coefficient between the estimates from different models and the measured BP. Bars represent the mean across all participants and each data point corresponds to one participant

Figure 5 shows that with only 15 min of data, NARX_physio_ achieves higher accuracy than NARX_30min_, with a MAE of 4.92 ± 5.7 mmHg for SBP and 4.4 ± 4.96 mmHg for DBP, compared to 5.77 ± 6.59 mmHg and 4.58 ± 5.54 m mmHg, respectively. Although not shown in Fig. 5a, ablation studies were conducted by removing the $\:MS{E}_{{\Delta\:}PPG}$ or $\:MS{E}_{PPG}$ terms from the loss function. These configurations resulted in higher MAE values compared to the full model (6.51 ± 8.05 mmHg for SBP and 5.56 ± 6.78 mmHg for DBP, and 6.68 ± 8.23 mmHg and 6.23 ± 7.20 mmHg, respectively), indicating that both components contribute to improved estimation performance. NARX_physio_ was the only model to exhibit almost significant (*p* = 0.009) better MAE then the MTD. No other statistical difference was observed du to low sample size.

The Pearson correlation coefficients (*r*) indicate better temporal alignment between the measured and estimated BP signals from the NARX_physio_ compared to the basic NARX_15min_, but may suffer from a small reduction in *r* for DBP estimates compared to NARX_30min_.

Figure 5 shows that the MTD model, which could be seen as a one-time cuff measurement, cannot capture the great variability of BP during daily life as reflected by the large σ_Err_. Table 1 shows a results summary across configurations and participants and their BP distributions during training and test. It can be observed that each participant’s σ_Err_ is always lower than their BP standard deviation during the test phase for the NARX_physio_.

**Fig. 6:**
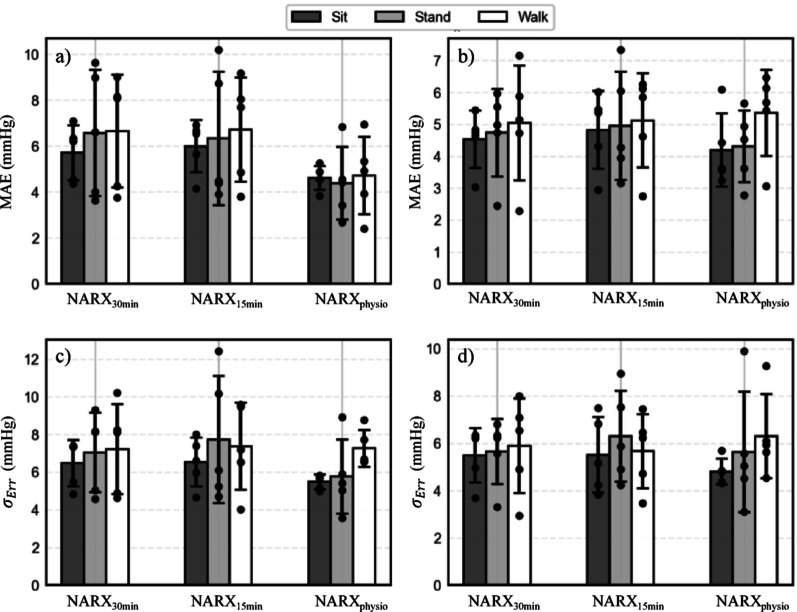
Comparison of model performance across activity conditions (sitting, standing, and walking) for the three models: NARX_30min_, NARX_15min_, and NARX_physio_. **a** and **b** show the mean absolute error (MAE) for SBP and DBP, respectively, while **c** and **d** show the corresponding standard deviation of the error (σ_Err_). Bars represent the mean across all participants and each data point corresponds to one participant

Model performance across activity conditions is shown in Fig. 6. Both MAE (Fig. 6a, b) and error variability (Fig. 6c, d) tend to increase from sitting to more active conditions. During sitting, the NARX_physio_ model exhibits reduced variability across participants, indicating more robust estimation in low-motion scenarios. Improvements with NARX_physio_ are more consistent for SBP, while DBP performance remains comparable across models.

**Table 1:** Training and test BP distribustions, and comparison of mean absolute error (MAE), mean error (μ_Err_), standard deviation of the error (σ_Err_), and Pearson correlation (r) coefficient between the estimates from different models for each participant

	Models	Training BP		Test BP		NARX_30min_				NARX_15min_				NARX_physio_			
	Metrics/subjects	μ	σ	μ	σ	MAE	μ_Err_	σ_Err_	*r*	MAE	μ_Err_	σ_Err_	*r*	MAE	μ_Err_	σ_Err_	*r*
SBP	P1	147.93	12.15	153.73	9.83	6.42	3.10	7.16	0.55	6.82	−4.59	7.13	0.48	3.89	−0.43	5.08	0.67
	P2	143.81	13.33	142.79	6.86	6.01	−2.96	7.15	0.63	5.80	−1.57	7.72	0.46	4.44	−1.36	5.89	0.48
	P3	131.12	10.33	135.22	7.74	4.90	−2.03	5.87	0.72	6.48	−5.43	5.70	0.60	5.22	−3.75	5.17	0.72
	P4	131.42	13.55	139.41	9.49	7.01	−4.88	7.43	0.60	7.14	−3.51	8.32	0.54	6.31	−3.72	6.86	0.52
	P5	113.90	8.30	114.18	7.61	4.20	−1.89	4.97	0.40	4.10	−2.09	4.67	0.42	4.76	2.88	5.49	0.43
DBP	P1	82.86	10.88	85.97	7.81	5.01	−0.70	6.53	0.47	6.05	−4.81	5.59	0.47	5.00	−3.80	4.88	0.52
	P2	80.16	10.66	79.46	6.24	4.52	1.03	6.04	0.48	5.30	0.77	7.31	0.36	4.32	−1.34	5.55	0.45
	P3	75.57	8.42	81.44	4.94	4.98	−3.55	5.11	0.56	4.31	−3.13	4.33	0.47	3.66	−1.56	4.38	0.49
	P4	76.08	10.04	84.10	6.21	5.45	−2.46	6.35	0.46	5.52	−1.71	7.01	0.42	5.52	−3.18	5.75	0.42
	P5	68.03	5.47	68.37	5.11	2.91	−1.17	3.68	0.55	2.99	0.23	3.93	0.41	3.49	1.57	4.25	0.37

**Fig. 7:**
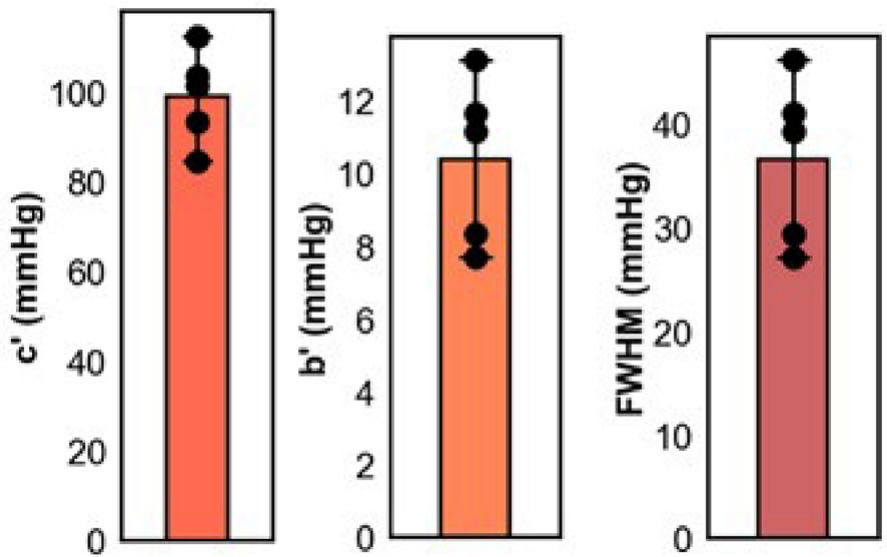
Mean and standard deviation of the *c’*, *b’* and FWHM parameters, exhibiting substantial inter-individual variability

Figure 7 shows the mean and standard deviation of the $\:{c}^{{\prime\:}}$, $\:{b}^{{\prime\:}}$ and FWHM parameters. As expected, these physiological parameters exhibit substantial inter-individual variability.

## Discussion

In this pilot study, we aimed at reducing the amount of data required for training subject-specific cuffless BP estimation models, which have the potential to overcome current accuracy limitations observed in cuffless devices, due to their tailored to the individual nature. While 15 min of training data is still a considerable amount, it may remain feasible for clinical use, particularly in scenarios requiring precise diagnostics—similar to 24-hour ambulatory BP monitoring. For example, a patient could be prescribed 24-hour cuffless BP monitoring session, beginning with a 15-minute calibration session using the clinic volume-clamping device. After this initial calibration, the patient could continue with their daily activities while wearing seamless ECG and PPG sensors, returning to the clinic after 24 h. The resulting continuous 24-hour estimated BP waveform could then be used for diagnostic purposes. Although scaling our findings from 5 participants over 6 h of daily activities to full 24‑hour monitoring will require substantial further work, this pilot study lays an important foundation for achieving that goal.

### Physiological Model

The proposed physiology-informed NARX model (i.e., NARX_physio_) leverages the known sigmoidal relationship between BP and PPG waveforms to enhance cuffless BP estimation. Compared to physics-informed neural network approaches that incorporate physiological constraints through loss function regularization, which has been applied to cuffless BP measurement [23], the proposed method embeds physiological knowledge directly into the model architecture via an explicit BP–PPG relationship. This structural integration enables the enforcement of physiological consistency during training while preserving a dynamic signal-based formulation. To our knowledge, this is the first demonstration of a dual‑output neural network architecture that integrates both physiological principles and the full BP waveform for improved estimation accuracy. NARX_physio_ consisted of two interconnected sub-models: a NARX-based model for BP estimation, and a physiological model that reconstructs PPG from the estimated BP. The latter includes a custom sigmoid-based layer with trainable subject-specific parameters designed to capture the nonlinear relationship between transmural pressure (BP – external pressure on an artery) and blood volume changes, as measured by the PPG signal [22]. In this study, transmural pressure was assumed to vary solely with BP, under the assumption of no external pressure variation on the PPG sensor. This allowed the model to rely exclusively on BP for the sigmoidal mapping, while the trainable *c* and *d* parameters accounted for the initial offset due to PPG contact pressure and PPG DC value, respectively.

The developed NARX_physio_ was specifically designed for subject-specific training, as the parameters of its sigmoidal function are tailored to each individual’s unique transmural pressure–blood volume relationship. This is reflected in Fig. 7, which shows substantial variability in the physiological parameters across individuals. The parameter *c’*, which shifts the sigmoid function to account for contact pressure, had an average value of 99 mmHg. Given that the PPG sensor was placed on the forehead, where BP is approximately 40 mmHg lower than at heart level due to hydrostatic pressure difference, this suggests an average contact pressure of 59 mmHg. This value is consistent with acceptable levels for signal quality and participant comfort.

The computed FWHM of the compliance curve $\:{g}^{{\prime\:}}$ was on average 36.5 mmHg. For comparison, we examined a previous study that used an exponential-linear function to model finger artery properties [22]. Using the derivative of that function, we found that the FWHM was approximately 33.4 mmHg on average for their participants. This comparison supports the physiological relevance of the sigmoid function used in NARX_physio_, indicating that it effectively captures a meaningful arterial compliance curve.

As briefly mentioned in the Methods Section, the parameter $\:d{\prime\:}$ reflects the DC component of the PPG signal, which lacks physiological significance. Similarly, $\:{a}^{{\prime\:}}$ represents the maximum blood volume in the artery, but only proportionally, as PPG measures volume in arbitrary units (volts). Therefore, $\:{a}^{{\prime\:}}$ also lacks direct physiological interpretation in this context.

### Model Performance

Herein, as a pilot evaluation of a physiology-informed BP waveform model, three model configurations were tested over a six-hour period and were compared to an oversimplistic MTD model reflecting a single cuff measurement in the morning to estimate a person’s BP. By linking the physiological model to the NARX model through shared dual-output loss functions during training, the architecture imposed a physiological constraint that reduced the amount of data required for training by 2. Although the BP–PPG relationship may vary across physiological states due to changes in vascular tone, the present approach models an average subject-specific mapping over the training period. This simplified representation, while not capturing state-dependent dynamics, appears sufficient to improve training efficiency and reduce data requirements, while preserving BP estimation performance. As shown in Fig. 5, NARX_physio_ even outperformed the standalone NARX_30min_ model in BP estimation with half the training data, which was already shown to outperform baseline PAT models [20]. Similar training data reduction and improved performance were reported by Sel et al. [23], where the physiology-informed part came from PPG features instead of enforcing a direct BP-PPG relationship.

The robustness of the proposed approach across different physiological and motion conditions is further illustrated in Fig. 6. The increased variability observed during standing conditions, similar to the walking condition, can be partly explained by the presence of sit-to-stand transitions, which induce rapid BP changes. These transitions constitute a substantial portion of the standing data despite the standing data representing a relatively small fraction of the overall dataset (≈ 7%), thereby disproportionately contributing to the observed variability. In contrast, sitting segments are more stable, which likely explains the reduced variability observed for the NARX_physio_ model under these conditions. Improvements associated with NARX_physio_ are more consistently observed for SBP, while DBP estimation remains comparable across models, suggesting that the physiological constraint primarily enhances robustness for SBP estimation.

Although subject-specific correlation coefficients between estimated and measured BP may appear modest (0.43–0.72 for SBP), this can be explained by the relatively small natural BP variability during daily life (standard deviation of 6.86–9.83 mmHg, Table 1) relative to measurement noise. Under such conditions, correlation coefficients are inherently constrained and may not fully reflect tracking performance. This interpretation is supported by prior work comparing cuff-based measurements: for example, Kallem et al. [25] reported noticeable discrepancies between two ambulatory BP monitoring (ABPM) devices over 8 h monitoring periods, illustrating that even reference measurements may exhibit limited agreement under low-variability conditions. Notably, on the same dataset used herein, simpler PAT-based models have been shown to yield substantially lower correlations (≈ 0.2–0.4 for SBP) [19], supporting the improved dynamic tracking achieved by the proposed approach. As illustrated in Fig. 2a–c, the model demonstrates high accuracy in tracking the full BP waveform, including key features such as DBP, SBP, and the dicrotic notch. However, during the Valsalva manoeuver (Fig. 2d), these features become more challenging to capture. While the model successfully tracks rapid BP changes of up to 30 mmHg within a few heartbeats, it tends to underestimate the dicrotic notches during this phase. This limitation is likely attributable to peripheral vasoconstriction induced by the manoeuver, which increases the viscoelastic properties of the arterial wall [26]. To address this, future improvements to the physiology-informed model will focus on incorporating a more advanced viscoelastic representation of the BP–PPG relationship [22].

An initial motivation for training NARX with data at both the beginning and the end of the monitoring period was to minimize drift [19]. This behavior is reflected in the NARX_15min_ model, which tends to exhibit a consistent negative bias across participants, whereas the NARX_physio_ model remains more centered around zero, suggesting improved stability over time (see Fig. 4). However, due to the limited duration of the dataset, the presence of longer-term drift (e.g., over 24 h) cannot be conclusively assessed. At the end of the 6-hour period, error values remain bounded for all models, with no evidence of divergence.

### PPG Estimation

The integration of PPG estimation may also offer practical benefits: real-time monitoring of PPG reconstruction error could serve as an indicator of physiological inconsistency. This error signal could be used to estimate model uncertainty, flag unreliable BP measurements, or even trigger model recalibration when necessary [27]. Such a self-calibration mechanism based on internal signal coherence could significantly enhance the reliability of wearable devices for continuous monitoring. This concept will be explored further in future work. Additionally, monitoring PPG contact pressure presents a promising avenue for improving model accuracy. In this study, variations in contact pressure likely occurred during daily activities, potentially changing the PPG signal and resulting in apparent BP changes. However, the true physiological transmural pressure variation may have come from a change in external pressure. Future work will incorporate direct measurements of PPG contact pressure to develop more accurate transmural pressure models that can account for and correct for these variations.

### Hyperparameter Tuning

In the initial model development phase, we conducted targeted hyperparameter exploration, not to exhaustively optimize the architecture, but to identify a configuration that produced consistently good performance across all five participants. For instance, several architectural variants were investigated, ranging from a low-depth network with a single hidden layer of 20 units to deeper architectures with three or four layers and up to 128 neurons. Larger networks occasionally improved training accuracy but tended to overfit, while very small ones failed to capture waveform dynamics. Among the tested activation functions, ReLU offered the most stable training, whereas hyperbolic tangent and exponential linear unit were less consistent. In terms of optimization, Adam offered faster and more stable convergence compared with stochastic gradient descent and NAdam. Using only MSE or MAE in the loss function was less effective than hybrid formulations. The most robust strategy combined MSE and MAE for BP values. For the NARX_Physio_ only, the MSE on the PPG waveform and its derivative resulted in the best results, where the derivative term preserved morphology and acted as a natural form of regularization. We initially tried conventional techniques, such as L2 regularization and dropout, but they provided limited benefit and often induced underfitting, resulting in a lower Pearson correlation coefficient between estimated and measured BP. Overall, these PPG physiology-inspired penalties stabilized training and ensured physiological consistency. This is supported by the ablation results, which show degraded performance when either the PPG reconstruction term or its derivative is removed, highlighting the complementary role of both components. We acknowledge that the optimal hyperparameters may shift as the approach is tested on a broader and more diverse population; thus, the present results should be viewed not as definitive but as a proof‑of‑concept demonstrating that physiologically informed structure can meaningfully improve performance over our baseline NARX architecture.

### Limitations

As mentioned on multiple occasion in this study, the main limitation is the small sample size, with data collected from only five participants. As a result, the findings may not fully capture the diversity of physiological responses across broader populations. Nevertheless, we chose to rely on this small dataset, as commonly available datasets that provide continuous BP waveforms over extended periods—such as MIMIC [28]—are collected in hospital settings and do not reflect the natural BP variations observed in real-world scenarios where hypertension is typically diagnosed. The IEEE Dataport dataset used herein, which includes six hours of daily living activities [21], was collected using a wearable volume-clamping device (Portapres; Finapres^®^ Medical Systems, Netherlands) that is no longer supported. This dataset is therefore particularly valuable, as current volume-clamping technologies typically require participants to remain tethered to a stationary system, limiting mobility during data collection. Nevertheless, certain extreme conditions—such as intense physical exertion or acute hypotension—and nighttime BP remain to be addressed using more challenging and varied datasets. It is worth noting that the NARX architecture was previously validated under moderate-and high-intensity exertion in another study [29]. However, we did not have access to those data and were therefore unable to evaluate the performance of the NARX_physio_ model under those conditions. Moreover, while the present work aims to reduce the amount of data required for subject-specific calibration, further reduction below 15 min is constrained by the limited BP variability achieved within the dataset protocol. The current fixed calibration protocol does not induce sufficiently rapid and diverse BP changes to support reliable training over shorter durations. A systematic evaluation of the minimum calibration duration would therefore require dedicated protocols specifically designed to elicit a broader range of hemodynamic variations.

It must be reiterated that subject-specific models may be viewed as a limitation to the generalizability of cuffless BP measurement. However, one can argue that they may represent the only viable approach to achieving clinically informative continuous BP monitoring. In this context, our work introduces a foundation model with the potential to substantially reduce the amount of data needed to train these subject-specific models. This may, in turn, make it feasible to collect the necessary data directly in the clinic prior to the 24-hour monitoring typically required for accurate hypertension diagnosis, which is generally performed using gold-standard but inconvenient ambulatory BP measurement [4].

## Conclusion and Future Work

In this pilot study, it was shown that a dual output physiology-informed NARX model maintained accurate BP estimation over a six-hour daily life period, when trained on half of the data of its non-physiology-informed counterpart. This was done by leveraging the intrinsic personalized four‑parameter BP–PPG sigmoidal layer into the neural network training process through a dual‑output loss function that simultaneously estimates the BP and PPG waveforms. Future work will focus on implementing a more advanced viscoelastic model of BP-PPG relationship, leveraging the estimated PPG signal for model recalibration and validating the model on a larger and more diverse population to ensure reliability across individuals.

## Data Availability

The data is available on IEEE DataPort under the name “Wearable Physiological and Blood Pressure Measurements During Activities of Daily Living”. The following reference can be use to refer to the dataset: Landry, C., Hedge, E. T., Hughson, R. L., Peterson, S. D. & Arami, A. Wearable Physiological and Blood Pressure Measurements During Activities of Daily Living. IEEE DataPort https://doi.org/10.21227/WYSP-GT69 (2021).Data can also be shared by the corresponding author (C.L.) on request.
